# Uric Acid and Potassium Serum Levels Are Independent Predictors of Blood Pressure Non-Dipping in Overweight or Obese Subjects

**DOI:** 10.3390/nu11122970

**Published:** 2019-12-05

**Authors:** Roberta Zupo, Fabio Castellana, Barbara Boninfante, Luisa Lampignano, Antonio Lattanzio, Rodolfo Sardone, Gianluigi Giannelli, Giovanni De Pergola

**Affiliations:** 1 Research Unit on Frailty Phenotypes, National Institute of Gastroenterology “Saverio de Bellis”, Research Hospital, Castellana Grotte, 70013 Bari, Italy; zuporoberta@gmail.com (R.Z.); castellanafabio@hotmail.it (F.C.); luisalampignano@gmail.com (L.L.); rodolfosardone@gmail.com (R.S.); 2 ISTAT (Italian Institute of Statistics), 70124 Bari Area, Italy; b.boninfante@libero.it; 3 Clinical Nutrition Unit, Medical Oncology, Department of Biomedical Science and Human Oncology, University of Bari, School of Medicine, Policlinico, 70124 Bari, Italy; antonio.lattanzio1996@gmail.com; 4 Scientific Direction, National Institute of Gastroenterology “Saverio de Bellis”, Research Hospital, Castellana Grotte, 70013 Bari, Italy; gianluigi.giannelli@irccsdebellis.it

**Keywords:** dipping, hypertension, potassium, uric acid, obesity

## Abstract

Background: Obesity and sleeping blood pressure (BP) abnormalities are well recognized as some of the main risk factors for hypertension and cardiovascular diseases (CVDs). The primary objective of this study was to evaluate the prevalence of hypertension and non-dipping profile in overweight/obese subjects. Methods: A sample of 100 consecutive healthy overweight/obese subjects, aged 20–69 years and never treated with antihypertensive drugs was examined. Ambulatory 24 h BP monitoring was performed to diagnose hypertension and a non-dipping profile. Anthropometric, metabolic and routine hematochemical parameters were assessed. All subjects underwent ultrasound measurement of common carotid intima–media thickness. Results: Hypertension was demonstrated in 69% (*n* = 69) and 27% of the sample (*n* = 27) had a non-dipping profile. Among the hematochemical variables, estimated glomerular filtration rate (eGFR) (*p* = 0.02) and FT4 (*p* = 0.01) serum levels were higher in the hypertensive group than in the normotensive group. Lower potassium and uric acid serum levels (*p* = 0.05) were independent predictive factors of a non-dipping BP profile. Conclusions: This study shows, for the first time, that 1) an unexpectedly high percentage (69%) of overweight/obese subjects is affected by hypertension; 2) early hypertensive subjects have an increased eGFR and higher FT4 serum levels; 3) lower potassium and uric acid levels are independent predictors of pathological nocturnal non-dipping.

## 1. Introduction

Obesity is a serious problem worldwide, since it is well known to significantly increase the risk of chronic diseases such as arterial hypertension, coronary heart diseases (CHDs), cardiovascular diseases (CVDs), type 2 diabetes, certain types of cancer, etc. [1]. Overweight and obesity are rapidly rising in most European countries, and recent estimates by the World Health Organization showed that 51.6% of the European population (18 years and over) has excess body weight (WHO, 2014). Several studies have shown a clear association between obesity and blood pressure (BP) [2], especially higher visceral fat, which is considered a triggering risk factor for hypertension [3,4]; by contrast, weight reduction is followed by a BP decrease, as well as the related complications [5]. It is well recognized that approximately 65%–75% of overweight and obese patients are at risk for hypertension [6] and that hypertensive subjects with obesity have a higher pulse pressure [7]. Among the mechanisms that link obesity to hypertension, the most relevant is the increased sympathetic activity in obese patients, and some authors have shown that the degree of sympathetic activation is higher in subjects with a central than those with a peripheral obesity phenotype [8]. Moreover, hyperinsulinemia is well known as a clinical feature frequently associated with obesity, and insulin indirectly contributes to hypertension by increasing sympathetic activity that, in turn, is responsible for an increased metabolic rate finalized to restore the perturbed energy balance in obesity [9]. Interestingly, sympathetic nervous system activation amplifies glomerular filtration, and this increase is regarded as an early precursor of organ damage in hypertensive patients [10]. In the renin-angiotensin–aldosterone system (RAS) [11], high circulating levels of leptin and additional abnormalities have also been identified, explaining the association between obesity and hypertension. In particular, leptin influences nitric oxide production and natriuresis and, along with chronic sympathetic activation, especially in the kidney, it may lead to sodium retention, systemic vasoconstriction, and BP elevation [12,13]. 

In recent decades, the use of ambulatory blood pressure monitoring (ABPM) has become widespread, since it has been recognized as a better predictor of major cardiovascular events than BP measurements in clinical settings. It helps to reduce the number of possible false readings and has the additional benefit of tracking the BP circadian rhythm. The latest guideline update for the clinical management of adult primary hypertension by the National Institute for Health and Clinical Excellence (NICE) recommends ABPM as gold standard to corroborate the diagnosis of hypertension in all adults with clinically elevated BP (NICE, 2011 National Institute for Health and Clinical Excellence). In accordance with the ACC/AHA 2017 guidelines, a normotensive patient should have a 24 h ABPM < 130/80 mmHg and this cut-off identifies the first stage of hypertension. Moreover, ABPM is the only method that can identify the non-dipping BP profile, which is defined as a nocturnal BP fall by less than 10%. Normally, BP follows a circadian rhythm, with 10%–20% lower values during sleep than during wakefulness. BP falls to its lowest during the first few hours of sleep (nocturnal dipping) and rises before awakening (morning surge). In some individuals, there is a blunted nocturnal increase in BP (non-dipping) [14]. This pathological pattern is mostly related to multifactorial pathogenic mechanisms such as sleep apnea syndrome, altered neurohormonal regulation (sympathetic nervous system, renin–angiotensin–aldosterone system) and extrinsic factors such as sodium load, sleep quality, physical daily inactivity and behavioral factors (smoking and alcohol intake) [15,16,17]. Several studies have suggested that a non-dipping BP profile is associated with subclinical organ damage and the incidence of cardiovascular outcomes [18].

Nowadays, a high percentage of obese subjects is not properly screened for hypertension, mainly because most overweight and obese patients do not contact their doctor until they develop symptoms and/or signs of hypertension; moreover, outpatient measurement itself is probably not sufficient. In addition, identifying some biomarkers that can predict non-dipper subjects may improve preventive tools. With this intent, we examined a cohort of 100 overweight subjects (body mass index (Body Mass Index) ≥ 25 Kg/m^2^) attending the Outpatients Clinic of Nutrition with the aim of losing weight and checked for daily BP and a dipping/non-dipping profile using ABPM. 

The main purpose of this study was to evaluate the prevalence of hypertension and non-dipping in a population of overweight and obese subjects. Secondly, we explored variables associated with non-dipping, such as hematochemical parameters and carotid intima–media thickness (c-IMT), measured as a tool to evaluate early atherosclerosis.

## 2. Methods

The study population was recruited from January 2018 to December 2018 at the Outpatients Clinic of Nutrition of the Medical Oncology Unit, Department of Biomedical Sciences and Human Oncology, University of Bari, School of Medicine, Policlinico, Bari, Italy, and at the National Institute of Gastroenterology "S. de Bellis," Research Hospital, Castellana Grotte, Italy. In total, 100 consecutive subjects were enrolled at the first examination if not taking any medication, including antihypertensive therapy, oral contraceptives or drugs for osteoporosis, and free of significant medical illnesses, except for overweight and obesity. Thus, the sample enrolled in this study consisted of 65 females and 35 males, aged between 20 and 69 years. Inclusion criteria were overweight or obesity (BMI > 25 Kg/m^2^ and < 40 Kg/m^2^) and presentation at the Outpatients Clinic with the sole aim of losing weight.

Exclusion criteria were a history of endocrinological diseases (diabetes mellitus, hypo or hyperthyroidism, hypopituitarism, etc.), chronic inflammatory diseases, stable known hypertension, angina pectoris, stroke, transient ischemic attack, heart infarction, congenital heart disease, malignancies, chronic inflammatory diseases, renal and liver failure, angina pectoris, myocardial infarction, heart failure, congenital heart diseases, minor and major stroke, inherited thrombocytopenias and other major diseases. 

At baseline, subjects were examined by means of medical history and anthropometric, metabolic and routine hematochemical parameters were assessed. The clinical physical exam included body weight, body mass index (BMI), and waist circumference (WC). Then, the subjects underwent 24 h BP measurement using ambulatory blood pressure monitoring (ABPM) in order to diagnose the possible presence of hypertension and of non-dipping. ABPM was measured at 15 min intervals from 07:00 to 11:00 and at 30 min intervals from 23:00 to 07:00 for 24 consecutive hours, starting from 08:30 (Ultralite ABPM Monitor 90217, SpaceLabs Media Inc, Redmond, WA). According to the latest ESH practice guidelines for ABPM, we applied fixed narrow time intervals with the aim of defining the wake-time BP and the sleep time BP periods (daytime defined as 09:00–21.00 h and night-time 01:00–06:00 h) [19]. Heart rate was measured over 24 h by the same instrument. The BMI was calculated by dividing the body weight (Kg) by the square body height (m^2^). Serum insulin concentrations were measured by radioimmunoassay (Behring, Scoppito, Italy). Serum 25(OH)D was quantified by a chemiluminescence method (Diasorin Inc, Stillwater, USA) and all samples were analyzed in duplicate. Plasma glucose was determined using the glucose oxidase method (Sclavus, Siena, Italy), while the concentrations of plasma lipids (triglycerides, total cholesterol, HDL cholesterol) were quantified by the automated colorimetric method (Hitachi; Boehringer Mannheim, Mannheim, Germany). LDL cholesterol was calculated by applying the Friedewald equation. Serum FT3, FT4 and TSH were measured using a competitive photometric method based on the solid phase antigen-linked technique (LIASON FT3, LIASON FT4, LIASON TSH, Dia-Sorin, Saluggia, Italy). Serum uric acid was measured by the URICASE/POD method implemented on an autoanalyzer (Boehringer Mannheim, Mannheim, Germany). Serum HbA1c was measured by high-performance liquid chromatography with coefficients of variations, 2% at low (5%) and high (10%) HbA1c values. HbA1c was standardized against the Diabetes Control and Complications Trial (DCCT) standard. Creatinine, sodium and potassium were measured by common laboratory methods. The MDRD 4 variable estimated glomerular filtration rate (eGFR) equation, which is based on a normalized body surface area (mL/min/1.73 m^2^), was used to calculate eGFR in our sample. Insulin resistance was assessed using the homeostasis model assessment (HOMA-IR) [20].

### Ultrasound Measurement of Carotid Intima–Media Thickness (c-IMT)

All subjects underwent high-definition vascular ultrasound according to the following protocol to measure c-IMT, a useful tool to evaluate early atherosclerosis. Ultrasonographic echo-color Doppler studies of the left and right common carotid arteries were performed by the same physician with a Philips Sonos 5500 using a 7.5 MHz high-resolution probe. The patients were placed in supine position, with the neck extended and rotated contralaterally by 45^°^, and the common carotid arteries were examined on the sagittal axis with a lateral view. We used the Pignoli et al. method to define c-IMT, as described in our previous studies [21]: by focusing and freezing images on the distal wall of the common carotid artery on the lengthwise axis during end-diastole, the c- IMT was calculated as the distance between the leading borders of the first hyperechoic line and of the second hyperechoic line, separated by a hypoechoic space (“double- track pattern”). The measurements were performed bilaterally 1 cm proximally to the carotid bulb, three times, and then the c-IMT value was calculated as the arithmetical mean of each side. The c-IMT value considered for statistical analyses was the mean of right and left measurements. For IMT detection, we excluded arterial segments presenting atherosclerotic plaque. 

The study protocol (ClinicalTrials.gov Identifier: NCT04093947) was approved by the Ethics Committee of the National Institute of Gastroenterology "S. De Bellis" Research Hospital, Castellana Grotte, Italy and all participants gave prior informed consent to enrolment in accordance with the Helsinki Declaration of 1964 and subsequent revisions.

We performed statistical analysis of baseline variables as the mean ± standard deviation (SD) for continuous variables and proportion (%) for the frequency of categorical variables. The correlation matrix was estimated for all the pairwise correlation coefficients between dipper/non-dipper and all variables examined (biochemical and anthropometric measurements). P-values less than or equal to 0.05 were considered statistically significant, with 95% confidence intervals. Variables with p-values lower than 0.05 at univariate analysis were included in a multivariate logistic model finalized to evaluate parameters independently related to modifications of the dipping profile. Statistical analysis was performed with RStudio software, Version 1.2.1335. Evaluation of the differences in mean values of the variables in the two different populations, dippers and non-dippers, and hypertensive and non-hypertensive, was carried out with Independent Samples t Tests or the Wilcoxon–Mann–Whitney test. A normal distribution was evaluated using the Shapiro test for each group in our sample, and specific parametric and non-parametric tests were performed to assess the presence of significant differences. The collected continuous variables were analyzed by Pearson’s linear correlation test (not shown) or Spearman’s rank correlation test, in accordance with the methodological occurrence.

## 3. Results

In total, 69% (*n* = 69) of our sample was diagnosed with hypertension (24 h ABPM ≥ 130/80 mmHg) and 27% (*n* =27) of the sample presented a non-dipping profile. After the diagnosis, we divided the whole sample into different groups, and compared the hypertensive versus normotensive group and dipping versus non-dipping group. Table 1 summarizes the differences in all the investigated variables between subjects with and without hypertension. As shown, eGFR (*p* = 0.02) and FT4 (*p* = 0.01) serum levels were higher in hypertensive than in normotensive subjects. In addition, we performed logistic regression models with the presence of hypertension as dependent variable and adjusting data for age, BMI and sex; this analysis showed that GFR is an independent predictive factor for hypertension (OR = 1.08 and CI = 1.02–1.14, *p* = 0.01). We applied the same logistic regression model to evaluate the relationship between hypertension and FT4 serum levels, adjusting for age, BMI and sex; this analysis showed an independent association between FT4 and hypertension (OR = 1.50 and CI = 1.00–2.26, *p* < 0.05). No substantial differences were found in the whole cluster of glycemic balance parameters (fasting blood glucose, HbA1c, insulin and HOMA-IR) between normotensive and hypertensive group. Table 2 summarizes the differences in all the investigated variables between dipping and non-dipping subjects. The non-dipping profile was observed in 27% (*n* = 27) of the sample and matched a lower daily and mean HR (*p*-value = 0.01 and 0.02, respectively). Furthermore, statistical analysis showed significantly lower potassium (4.18 mEq/l) and uric acid (4.22 mg/dl) levels in the group of non-dipper subjects (*p* < 0.05). A multivariate logistic regression model was built to evaluate the relationship between the dipping and non-dipping pattern, where non dipping was the dependent variable and potassium and uric acid were covariates in two separate models, both adjusted for age, sex and BMI. Potassium and uric acid seem to be good predictors of a non-dipping profile (OR = 0.16, CI = 0.03–0.99, *p* < 0.05 and OR = 0.55, CI = 0.34–0.90, *p* < 0.05, respectively). We created a new covariate, resulting from the product of uric acid and potassium, to test the interaction effect of uric acid on the association of potassium with the dipping profile (OR = 0.82, CI = 0.69–0.97, *p* = 0.02.) The model was designed to show the effect of the interaction between uric acid and potassium serum levels in predicting a non-dipping profile. The interaction range of the combined variable was greater than the sum of both variables, showing the role of the interaction in predicting hypertension. 

## 4. Discussion

The main goal of the present study was to investigate the prevalence of hypertension within a sample of apparently healthy subjects but overweight or obese (BMI ≥ 25 Kg/m^2^), taking no medication and not diagnosed with stable hypertension. Hypertension was diagnosed by ABPM following the latest ACC/AHA 2017 guidelines, and we found that 69 subjects (69% of the whole sample) had hypertension, previously undiagnosed. This finding is particularly interesting and clinically important, since it suggests that hypertension is commonly diagnosed late in most obese subjects; this phenomenon may be responsible for accelerating complications induced by hypertension in obese subjects. 

It is well known that obesity is a primary cause of both essential hypertension and kidney dysfunction. In fact, obesity increases tubular reabsorption, which consequently shifts pressure natriuresis towards higher BP values [22]. The mechanisms responsible for increased sodium reabsorption and altered pressure natriuresis in obesity include activation of the renin–angiotensin system (RAS) and sympathetic nervous system, and physical compression of the kidneys, which is related to the accumulation of intrarenal fat and extracellular matrix. In this regard, we previously showed a positive association between para- and perirenal fat accumulation and higher 24 h mean systolic and diastolic BP levels in overweight and obese subjects [23]. Accordingly, glomerular hyperfiltration, which is demonstrated by an increased eGFR, is a form of compensatory mechanism for both the increased renal tubular reabsorption and impaired sodium balance in the early phases of obesity [10].

The second interesting finding in this study is that hypertension was correlated with increased FT4 serum (*p* = 0.01) and eGFR (*p* = 0.02) serum levels, independently of sex, BMI and age. Moreover, we found higher levels of FT4 in the hypertensive than in the normotensive group. On the basis of these results, we may hypothesize that hypertension is characterized in obesity by a decreased activity of thyroxine 5-deiodinase, converting FT4 to FT3, possibly due to insulin resistance [24]. Concerning the higher eGFR in our hypertensive subjects, it is well known that obesity is characterized by hyperfiltration and preglomerular vasodilation, which progressively decreases with advancing age [25]. It may well be that the appearance of hypertension is associated with an even higher eGFR than in subjects with obesity and without hypertension. Concerning the mechanism associating obesity higher eGFR and hypertension, according to the suggestion of Hall et al [26], it may well be that, initially, obesity causes renal vasodilation and glomerular hyperfiltration, which act as compensatory mechanisms to maintain sodium balance despite increased tubular reabsorption. However, these compensations, along with increased arterial pressure and metabolic abnormalities, may ultimately lead to glomerular injury and initiate a slowly developing vicious cycle that exacerbates hypertension and worsens renal injury.

We assessed the prevalence of the non-dipping pattern among overweight and obese individuals and found that 27% of these subjects were non-dipping, meaning that more than one subject among four individuals with a high BMI has an impaired BP physiological chronobiology. This is an important point since non-dipping subjects are well known to be at increased cardiovascular risk [27] and more likely to have target organ damage, including silent cerebrovascular and kidney injury [28]. Interestingly, we also showed that lower potassium serum levels were predictive of pathological dipping (*p* = 0.05). This is a very new finding since, to date, the activity of the sympathetic nervous system, the level of exercise, arousal during the day and the night, the depth and quality of sleep, etc., have been described as the main pathophysiological mechanisms involved in the shift from a dipping to a non-dipping pattern [29,30]. 

Some authors have considered the non-dipping pattern related to an impaired capacity to excrete sodium during daytime [31]. Consequently, to maintain the 24 h sodium balance, BP increases at night to promote sodium excretion. Potassium and sodium are closely interconnected but have opposite effects in the body. A high sodium intake increases BP, which can lead to heart disease, while a high potassium intake can help to relax blood vessels and excrete sodium, decreasing the BP. Wilson et al. were the first to show that dietary potassium supplementation, as well as sodium restriction, could restore normal dipping without having any effect on daytime BP, and this effect was stronger in salt-sensitive subjects [32]. In line with this, our data show that lower potassium serum levels are a predictive parameter in the setting of the physiological nocturnal BP fall. Moreover, considering our study population conditions of overweight or obesity, it seems important to take into account also lifestyle factors such as dietary habits. Consuming too much sodium and not enough potassium is responsible for a raised BP. The majority of sodium intake comes from dietary processed foods, which are to be classified as products indicating unhealthy dietary habits. A healthy diet features a high intake of fruits and vegetables, which are the main sources of potassium. Among these, the leading foods for potassium content are potatoes, mushrooms, clams, lentils, white soybean and bananas [33]. Thus, it is likely that wrong dietary habits also play a role in decreasing potassium serum levels.

Lastly, our data showed that compared to dipping subjects, non-dipping subjects had lower mean uric acid levels. This result is not in line with the literature evidence, especially when we consider that our sample included overweight subjects. In any case, it is noteworthy that acid uric itself has strong antioxidant properties and may act as an immune system stimulant. At physiological concentrations, urate reduces the oxo-heme oxidant formed by peroxide reaction with hemoglobin and protects both erythrocyte ghosts against lipid peroxidation and erythrocytes from peroxidative damage leading to lysis [34]. In contrast, high urate levels are a well-known strong risk factor for gout and renal calculi, as well as a triggering factor for metabolic syndrome and CVDs [35]. The lowest mean levels of urate found in the non-dipping group suggested that people who have this pathological pattern also have one less protective factor, if we consider uric acid useful for its antioxidant effects. However, having lower uric acid levels in non-dipper subjects is not definitively an unfavorable result, since even normotensive subjects also had lower mean values of uric acid.

IMT was not significantly different between the hypertensive and normotensive groups or between subjects with and without dipping, suggesting that (1) subjects with higher BP were early hypertensive subjects who had not yet developed early atherosclerosis and that (2) non dipping is not so severe an alteration as to increase the thickening of the arterial wall.

Interestingly, the BMI and waist circumference were not different between subjects with or without hypertension, suggesting that obesity per se, more than the level of obesity or the body fat distribution, is responsible for the onset of hypertension and a non-dipping profile. Moreover, gender did not seem to influence the percentage of hypertension or non-dipping profile in our population.

Since the grade of obesity and insulin levels were not different between subjects with or without hypertension and between dipper and non-dipper subjects, it may well be that genetic and hemodynamic factors are more important than body fat and metabolic parameters in determining the development and the characteristics of hypertension in obesity.

Apparent limitations of the study are the relatively small sample size and the higher prevalence of women in the population under study. Concerning the latter point, a clear explanation is that the recruitment took place at the Outpatients Clinic of Nutrition, where subjects are educated to eat in a healthier way and to check their body weight and it is well known that women are more interested than men from this point of view. Third, we did not measure markers of inflammation or oxidation, which could have helped us to better understand some findings and, in particular, those concerning uric acid associations. Fourth, it should be stressed that the concept that the day–night BP changes and the classification of patients into dippers and non-dippers are poorly reproducible over time. For the future, in order to improve the accuracy of the measurement, the quality and the duration of sleep, the use of actigraphy and polysomnography should be considered. By contrast, a strong aspect of this study is that we examined only individuals who were not taking any medication and, therefore, there was no interference of drugs on the statistical associations. Nevertheless, our data on a small sample leave room for further investigations to extend this research, for example, hormonal essays taking into account the possible role of aldosterone, already described as being involved in a non-dipping pattern.

## 5. Conclusions

This study shows that the majority of obese people remain undiagnosed for hypertension for a long time, facilitating the onset of hidden complications induced by hypertension itself. Moreover, approximately one-quarter of them present a non-dipping BP profile, which is commonly associated with an increased cardiovascular risk. Lower potassium and uric acid serum levels seem to be predictive of a pathological impairment of BP chronobiology.

## Figures and Tables

**Table 1:** Differences in baseline variables between hypertensive and non-hypertensive subjects.

Variables	Non-Hypertensive	Sd	Hypertensive	Sd	*p*-Value *
**Gender %**	M = 8; F = 23		M = 27; F = 42		0.28
**Age (y)**	46.6	12.5	44.7	9.79	0.41
**Body mass index (BMI) (Kg/m^2^)**	32.37	5.85	32.56	4.54	0.47
**Waist circumference (WC) (cm)**	106.00	10.98	108.51	11.75	0.32
**Intima-media thickness (IMT) (mm)**	0.72	0.15	0.70	0.13	0.42
**Mean heart rate (bpm)**	75.2	8.99	76.6	8.12	0.45
**Day time heart rate (bpm)**	78.3	9.64	79.3	8.79	0.6
**Night time heart rate (bpm)**	66.8	8.53	69.1	8.90	0.27
**Potassium (mEq/L)**	4.36	0.27	4.28	0.38	0.33
**Sodium (mmol/L)**	141.73	2.10	140.25	2.70	0.06
**Uric acid (mg/dl)**	4.28	1.15	4.75	1.52	0.14
**HDL cholesterol (mg/dl)**	54.83	15.53	50.07	13.93	0.13
**Triglycerides (mg/dl)**	111.67	44.85	126.22	74.83	0.7
**Total cholesterol (mg/dl)**	210.53	44.41	204.64	39.42	0.76
**LDL cholesterol (mg/dl)**	131.83	38.16	132.23	35.41	0.93
**Fasting blood glucose (mg/dl)**	90.90	9.77	91.26	11.17	0.96
**Insulin (mg/dl)**	16.69	10.07	16.49	8.91	0.9
**HOMA-IR**	3.79	2.42	3.73	2.07	0.86
**HbA1c (%)**	5.46	0.42	5.39	0.33	0.48
**GFR (mL/min)**	94.33	16.99	104.32	14.76	0.02 *
**Creatinine (mg/dl)**	0.78	0.17	0.77	0.15	0.82
**Vitamin D (ng/mL)**	20.55	7.80	20.07	8.06	0.46
**TSH (mU/L)**	2.38	1.18	2.06	0.94	0.15
**FT3 (pg/mL)**	3.06	0.37	3.06	0.37	0.99
**FT4 (pg/mL)**	10.14	1.04	10.65	1.24	0.01 *

* Independent Samples t-Test.

**Table 2:** Differences in baseline variables between dipper and non-dipper subjects.

Variables	Dippers	Sd	Non-Dippers	sd	*p*-Value *
**Gender %**	M = 24; F = 49		M = 11; F = 16		0.62
**Age (y)**	48.25	10.96	44.16	9.35	0.09
**BMI (Kg/m^2^)**	32.74	4.94	31.85	5.03	0.37
**24 h SBP (mm/Hg)**	130.38	9.99	131.93	12.87	0.53
**Day SBP (mm/Hg)**	135.97	9.74	133.04	12.66	0.22
**Night SBP (mm/Hg)**	115.27	11.71	128.89	14.63	5.33e-6 **
**24 h DBP (mm/Hg)**	81.96	7.20	84.37	9.86	0.25
**Day DBP (mm/Hg)**	86.46	7.54	85.59	9.57	0.64
**Night DBP (mm/hg)**	69.64	7.57	80.85	11.60	4.11e-5 **
**IMT (mm)**	0.70	0.12	0.72	0.18	0.53
**Mean heart rate (bpm)**	77.33	8.04	72.93	8.57	0.02 **
**Day heart rate (bpm)**	80.43	8.66	75.19	9.03	0.01 **
**Night heart rate (bpm)**	68.71	8.78	67.41	8.99	0.51
**Mean pulse pressure (mm/Hg)**	48.42	7.62	47.56	5.59	0.59
**Potassium (mEq/l)**	4.36	0.34	4.18	0.32	0.05 **
**Sodium (mmol/L)**	140.51	2.59	141.37	2.52	0.23
**Uric acid (mg/dl)**	4.74	1.54	4.22	0.95	0.05 **
**TSH (mU/L)**	2.14	1.04	2.20	1.00	0.77
**FT3 (pg/mL)**	3.07	0.38	3.03	0.35	0.66
**FT4 (pg/mL)**	10.61	1.19	10.18	1.19	0.12
**WC (cm)**	108.49	12.15	105.67	9.54	0.23
**Triglycerides (mg/dl)**	121.04	72.94	123.85	50.24	0.83
**Total cholesterol (mg/dl)**	205.31	42.56	209.41	36.50	0.64
**HDL cholesterol (mg/dl)**	52.33	15.30	49.33	12.20	0.27
**LDL chol (mg/dl)**	130.50	37.16	136.41	33.26	0.29
**FBG (mg/dl)**	90.45	9.83	93.04	12.81	0.37
**Insulin (mg/dl)**	16.75	8.69	16.03	10.71	0.4
**HOMA-IR**	3.79	2.11	3.63	2.37	0.6
**HbA1c (%)**	5.38	0.37	5.49	0.33	0.23
**GFR (ml/min)**	101.57	17.69	100.44	11.53	0.76
**Adrenalin(µg/24 h)**	6.43	4.60	5.77	5.19	0.39
**Noradrenalin (µg/24 h)**	33.86	17.05	35.20	20.45	0.78
**Aldosterone (ng/dl)**	15.67	7.9	16.22	5.3	0.44
**Creatinine (mg/dl)**	0.77	0.16	0.78	0.17	0.73
**Vitamin D (ng/mL)**	20.26	8.41	20.13	6.86	0.95

* Independent Samples t-Test; ** Wilcoxon-Mann-Whitney test.

## Abbreviations

BP	Blood pressure
ABPM	ambulatory blood pressure monitoring
c-IMT	common carotid intima–media thickness
BMI	body mass index
WC	waist circumference
HOMA	homeostasis model assessment
CVDs	cardiovascular diseases
eGFR	estimated glomerular filtration rate
